# A draft genome sequence of *Cellulomonas* sp. ICMP 17802, isolated from root nodules of *Vicia faba*

**DOI:** 10.1128/mra.00899-24

**Published:** 2024-11-27

**Authors:** Josie C. Mainwaring, Monica L. Gerth

**Affiliations:** 1 School of Biological Sciences, Victoria University of Wellington, Wellington, New Zealand; University of Strathclyde, Glasgow, United Kingdom

**Keywords:** CAZyme, CAZome, cellulolytic enzymes, DNA sequencing, ICMP

## Abstract

Here, we report the draft genome sequence of *Cellulomonas* sp. ICMP 17802. This bacterium was obtained from the International Collection of Microorganisms from Plants and was originally isolated from root nodules of *Vicia faba*. The draft genome contains 154 predicted carbohydrate-active enzyme genes.

## ANNOUNCEMENT

*Cellulomonas* species (1) are well-studied for their cellulase-producing capabilities (2–4). Bacterial cellulases have diverse applications across multiple industries (5), including food and fermentation (6, 7), textiles and detergents (8, 9), pulp and paper (10), and the valorization of lignocellulosic biomass (11). Our lab is investigating cellulase-producing microorganisms as biological control agents for oomycete diseases (12).

*Cellulomonas* sp. ICMP 17802 was isolated from *Vicia faba* root nodules in 2008 and stored in liquid nitrogen at the International Collection of Microorganisms from Plants (ICMP). We requested the isolate in 2021; it was revived onto Reasoner’s 2A agar and transferred to storage agar (yeast extract 3.0 g/L, NH_4_Cl 0.5 g/L, KCl 0.2 g/L, MgSO_4_.7H_2_O 0.2 g/L, K_2_HPO_4_ 1.0 g/L, and agar 15 g/L) for shipping. Upon arrival, the isolate was cultured in tryptic soy broth at 28°C with 200 rpm shaking before storing in 25% glycerol at −70°C. *Cellulomonas* sp. ICMP 17802 was revived onto tryptic soy agar in 2022, and a single colony was cultured in tryptic soy broth at 25°C with 200 rpm shaking for genomic DNA extraction. We pelleted approximately 10^8^–10^10^ cells (per extraction) in microcentrifuge tubes and extracted the genomic DNA using a Monarch Genomic DNA Purification Kit (New England Biolabs) following the manufacturer’s protocol for Gram-positive bacteria.

The *Cellulomonas* sp. ICMP 17802 genome was sequenced using Illumina short-read sequencing by SeqCenter (Pittsburgh, PA). Sample libraries were prepared using the Illumina DNA Prep kit and IDT 10 bp unique dual indices and sequenced on an Illumina NextSeq 2000, producing 2 × 151 bp reads. Demultiplexing, quality control, and adapter trimming were performed with bcl-convert v3.9.3. Assembly was performed using Unicycler v0.4.8 (13), and assembly statistics were evaluated using QUAST v5.0.2 (14). Genome annotation was done through the NCBI Prokaryotic Genome Annotation Pipeline v6.7 (15) as part of the GenBank submission process. Carbohydrate-Active enZyme (CAZyme) genes were predicted using the agreement of at least two of the three tools used by run_dbcan v4.1.4 (16): “hmmer,” a HMMER (v3.4) search against the dbCAN CAZyme domain hidden Markov model (HMM) database; “dbsub,” a HMMER (v3.4) search against the dbCAN-sub HMM database; and “diamond,” a DIAMOND (v2.1.9) search against the CAZy database (17). Default parameters were used for all software unless otherwise specified.

Genome features for *Cellulomonas* sp. ICMP 17802 are summarized in Table 1. The genome contains 154 predicted CAZymes, with glycoside hydrolases comprising the majority (59%). Many of these belong to families involved in hemicellulose hydrolysis (Additional file 1). Taxonomic assignment was performed using GTDB-Tk v2.3.2 (18), which placed the query genome into the Genome Taxonomy Database (GTDB; release 07-RS207) (19) bacterial reference tree using the maximum-likelihood placement tool pplacer (v1.1.alpha19) (20) and calculated average nucleotide identity (ANI) using FastANI (v1.34) (21). The GTDB-Tk tree topology confirmed the assembly’s placement within the genus *Cellulomonas*, but the ANI of the query genome compared to the *Cellulomonas* reference genomes in the GTDB was lower than the 95% threshold for species assignment. These results suggest that *Cellulomonas* sp. ICMP 17802 represents a previously undescribed species within the genus *Cellulomonas*.

**TABLE 1.** Assembly statistics and genome information for *Cellulomonas* sp. ICMP 17802

Parameter	Finding for *Cellulomonas* sp. ICMP 17802
Sequencing and assembly	
No. of Illumina reads	7,422,930
Total no. of Illumina bases	1,216,097,586
Avg coverage (×)	303
Total no. of contigs	59
Largest contig (bp)	732,310
*N*_50_	116,811
*L*_50_	9
Genome description	
Genome size (bp)	4,008,555
G + C content (%)	73.5
Total no. of genes	3,824
Total no. of coding sequences	3,774
No. of proteins	3,763
No. of pseudogenes	11
Total no. of RNAs	50
No. of rRNAs	3
No. of tRNAs	44
No. of non-coding RNAs	3

## Data Availability

All data can be found under the NCBI BioProject accession number PRJNA1141150, including sampling information (BioSample accession number SAMN42882187), raw reads (SRA accession number SRR30166926), and genome assembly information (GenBank accession number JBGFQZ000000000; the version described in this paper is version JBGFQZ000000000.1). The predicted CAZyme genes in the *Cellulomonas* sp. ICMP 17802 assembly (Additional file 1) are available at Figshare (https://doi.org/10.26686/wgtn.26759968). Information on the original sampling and subsequent storage of *Cellulomonas* sp. ICMP 17802 is available at https://scd.landcareresearch.co.nz/Specimen/ICMP_17802.
